# Blurring the Lines: Co‐Occurrence of MSH6 Variant and MLH1 Constitutional Epimutation in a Young Colorectal Cancer Patient

**DOI:** 10.1111/cge.70045

**Published:** 2025-08-09

**Authors:** Aasem Abu Shtaya, Yarin Hadid, Ahmad Mahamid, Debora Kidron, Naama Halpern, Adel Shalata, Zohar Levi, Yael Goldberg

**Affiliations:** ^1^ Recanati Genetics Institute Rabin Medical Center—Beilinson Hospital Petach Tikva Israel; ^2^ Unit of Gastroenterology Lady Davis Carmel Medical Center Haifa Israel; ^3^ Bnei Zion Medical Center Simon Winter Institute for Human Genetics Haifa Israel; ^4^ Department of Surgery Carmel Medical Center Haifa Israel; ^5^ The Ruth and Bruce Rappaport Faculty of Medicine, Technion Haifa Israel; ^6^ Department of Pathology Meir Hospital Kfar Saba Israel; ^7^ Gray Faculty of Medical and Health Sciences Tel Aviv University Tel Aviv Israel; ^8^ The Jusidman Cancer Center Sheba Medical Center Ramat Gan Israel; ^9^ Division of Gastroenterology Rabin Medical Center—Beilinson Hospital Petach Tikva Israel

**Keywords:** cancer, colorectal, constitutional epimutation, lynch, methylation, MLH1, MSH6

## Abstract

Lynch syndrome (LS) is an autosomal dominant hereditary cancer predisposition syndrome caused by germline pathogenic variants in DNA mismatch repair (MMR) genes. We report a 27‐year‐old woman with right‐sided colorectal cancer, café‐au‐lait macules, and an occipital neurofibroma. Tumor testing revealed microsatellite instability, loss of MLH1 and PMS2 expression, high tumor mutational burden (21.87 mutations/Mb), and wild‐type *BRAF*. Germline analysis revealed a heterozygous *MSH6* pathogenic variant inherited from her father. Additionally, *MLH1* promoter hypermethylation was detected in peripheral blood DNA, consistent with constitutional mosaic epimutation. Constitutional epigenetic silencing of *MLH1* is a rare but established cause of LS. This is the first reported case of a dual mechanism involving both a germline *MSH6* variant and constitutional *MLH1* methylation. The patient's unique clinical presentation and molecular profile challenged the conventional binary classification of MMR deficiency as either hereditary or sporadic, and highlight the complexity of MMR‐related tumorigenesis. This case underscores the importance of comprehensive assessment integrating tumor and germline molecular data, particularly when clinical or molecular findings are atypical or discordant. The digenic etiology also raises questions regarding cancer surveillance and management strategies in such individuals.

## Graphical abstract

We report a unique case of early‐onset colorectal cancer with both a germline *MSH6* variant and constitutional mosaic *MLH1* epimutation, revealing a possible digenic mechanism underlying Lynch syndrome. This case highlights the diagnostic complexity of mismatch repair deficiency and the value of integrative tumor–germline molecular profiling.

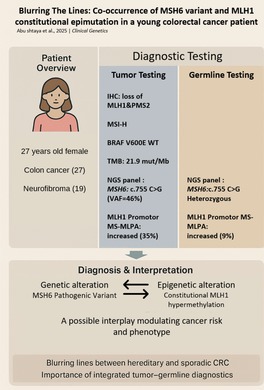

## 1 Introduction

Lynch syndrome (LS) is an autosomal dominant hereditary cancer syndrome caused by germline pathogenic variants (PVs) in the mismatch repair (MMR) genes *MLH1*, *MSH2*, *MSH6*, and *PMS2* [1]. These genes play a role in maintaining genomic stability by correcting DNA replication errors. Approximately 1.5%–4% of LS cases are caused by epigenetic events such as *MSH2* silencing due to *EPCAM* deletions or *MLH1* promoter hypermethylation [2, 3, 4].

Loss of MMR function leads to microsatellite instability (MSI), a hallmark of LS, which significantly increases the risk of colorectal, endometrial, and additional extracolonic malignancies [3]. Universal screening strategies, performed via immunohistochemistry (IHC) or MSI testing, have become a standard practice in identifying patients at risk for LS [5]. Loss of *MLH1* and/or PMS2 expression is caused by either: (i) germline PVs, (ii) somatic *MLH1* promoter hypermethylation, (iii) biallelic somatic *MLH1* mutations, or rarely (iv) constitutional *MLH1* hypermethylation [6]. In MMR‐deficient colorectal cancer (CRC), testing for *MLH1* methylation and BRAF V600E mutation is warranted to distinguish sporadic cancers from LS‐associated tumors [7].

Constitutional epimutations refer to epigenetic abnormalities present in normal tissues. Constitutional *MLH1* promoter methylation results in allele silencing [7]. These epimutations are classified as primary (non‐Mendelian, reversible) or secondary (linked to cis‐acting variants, inherited dominantly). Distinguishing between them is key for genetic counseling [6].

Emerging evidence supports a role of digenic inheritance—where PVs in two cancer genes co‐occur and contribute to atypical LS phenotypes [8]. Additionally, *MLH1* promoter methylation has been reported in a subset of *MLH1* variant carriers, implying that methylation and mutation are not mutually exclusive [9]. This highlights the importance of integrating tumor and germline profiling when needed to discern sporadic from hereditary tumors [10].

We report a patient with early‐onset CRC, harboring a germline *MSH6* PV and constitutional *MLH1* epimutation. The aim of this report is to present a unique case of early‐onset colorectal cancer characterized by the rare co‐occurrence of a germline *MSH6* PVs and constitutional *MLH1* promoter hypermethylation, highlighting the implications of digenic mechanisms in LS diagnostics and management.

## 2 Materials and Methods

### 2.1 Data Collection

The relevant demographic, clinical, pathologic, and genetic data were retrieved from the medical file. The study was approved by the institutional review board of Rabin Medical Center (code 0847–22).

### 2.2 Germline Genetic Analysis

Testing was performed on a clinical basis, with the patient's consent to join a research cohort. The study was approved by the institutional Research Ethics Committee. A comprehensive Next Generation Sequencing (NGS) analysis of 13 (*APC*, *BMPR1A*, the 3′‐untranslated region [UTR] of *EPCAM*, *MLH1*, *MSH2*, *MSH6*, *MUTYH*, *PMS2*, *PTEN*, *SMAD4*, *STK11*, *TP53*) hereditary cancer predisposition genes was performed on the patient's whole blood. The test analyzed single nucleotide variants (SNVs), small insertions and deletions (InDels), and copy number variations (CNVs) in the coding regions and splicing sites. The sequencing FastQ data were analyzed by Sophia DDM platform (Sophia Genetics) and Franklin platforms (GENOOX). Methylation analysis was performed through Methylation‐specific multiplex ligation‐dependent probe amplification (MS‐MLPA) assay (ME011‐B3, MRC Holland, Amsterdam, Netherlands) on the patient's blood according to the manufacturer's instructions, and analysis was conducted using Coffalyser software (MRC‐Holland).

### 2.3 Tumor Genetic Analysis

Tumor and non‐malignant colonic tissue were tested for MMR protein expression by IHC measured by Ventana Abs, using a Ventana immunostainer. *BRAF* V600E mutation was evaluated by PCR using Idylla tests.

Tumor molecular profiling was carried out on the Ion Torrent platform, using the Oncomine Comprehensive assay. It includes MSI testing, tumor mutation burden (TMB) measurement, and a comprehensive NGS‐based assay, including sequencing of DNA and RNA of 500 cancer‐related genes. Tumor methylation was assessed using the MS‐MLPA assay.

## 3 Results

### 3.1 Clinical Description

A 27‐year‐old woman presented with recurrent rectal bleeding and abdominal pain. Her medical history was notable for a resected scalp neurofibroma at age 19. She reported no other significant medical or surgical conditions. A colonoscopy identified a polypoid lesion in the right colon. Biopsy revealed moderately differentiated adenocarcinoma. Staging with whole‐body CT showed no evidence of distant metastasis, and carcinoembryonic antigen levels were within normal range. She underwent a right hemicolectomy. Pathological assessment confirmed adenocarcinoma invading the muscularis propria (pT2), with one of 43 lymph nodes positive for metastatic carcinoma (pN1). She was diagnosed with stage III CRC and received adjuvant chemotherapy with capecitabine and oxaliplatin.

On physical examination, five café‐au‐lait macules (CALMs) were noted. MRI of the left posterior thigh demonstrated an arteriovenous malformation (AVM) corresponding to mild swelling in that region.

**FIGURE 1:**
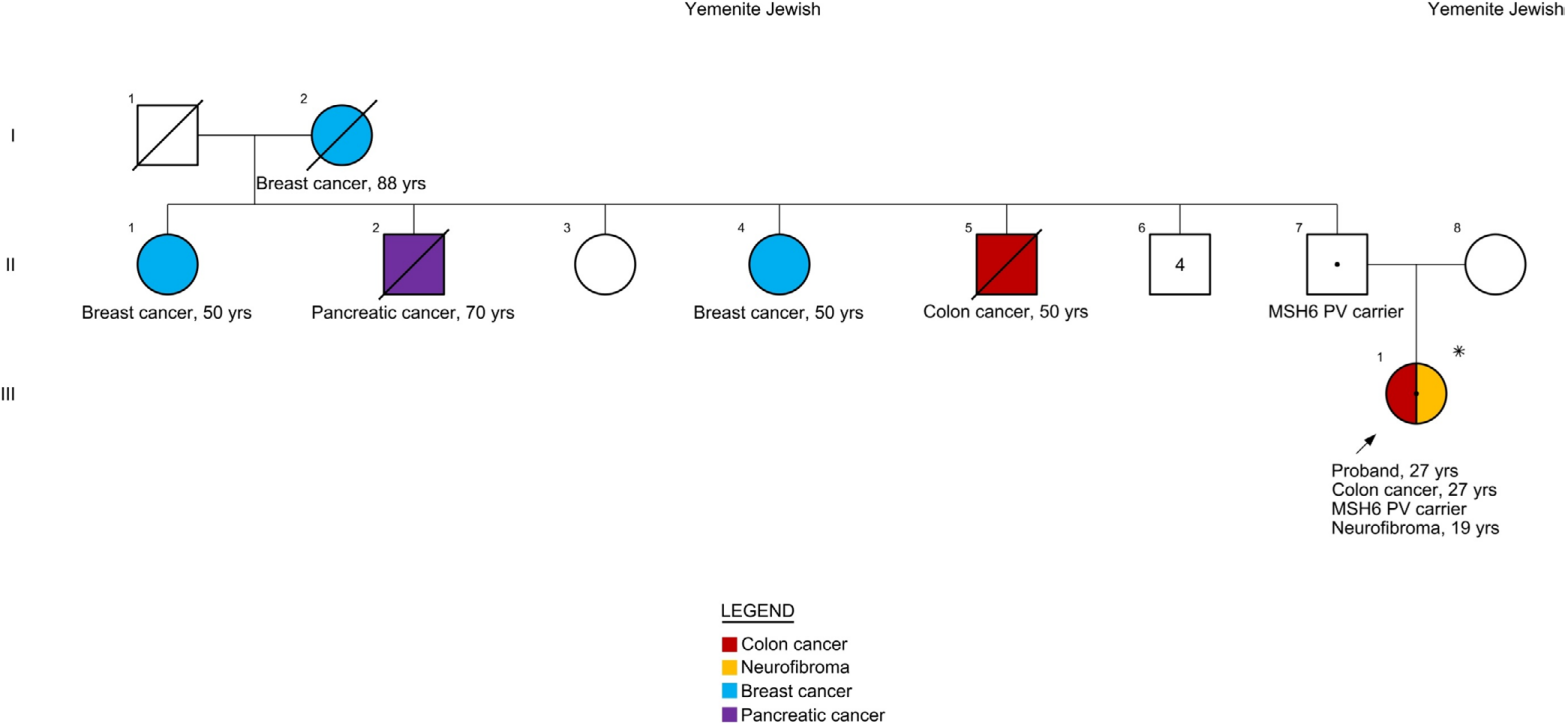
Family pedigree showing affected and healthy members along with *MSH6* variant carriers.

Both parents were clinically unaffected. Her mother had a history of two tubular adenomas detected on routine colonoscopy. The father's colonoscopy was unremarkable. Paternal family history was notable for cancer: a paternal uncle with CRC at age 50, another paternal uncle with pancreatic cancer at age 70, and two paternal aunts with breast cancer diagnosed at age 50 (Figure 1).

### 3.2 Tumor Molecular Findings

IHC for the MMR proteins revealed complete loss of nuclear expression on MLH1 and PMS2 with intact staining of MSH2 and MSH6. Surrounding non‐neoplastic tissue showed preserved expression of all four MMR proteins, serving as an internal positive control.

Tumor molecular profiling demonstrated high MSI and high TMB of 21.87 mutations per megabase. No *BRAF* V600E mutation was detected, consistent with a wild‐type *BRAF* status. NGS of the tumor using a comprehensive cancer gene panel identified a truncating PV in *MSH6* (c.755C>G; p.Ser252Ter) with a variant allele frequency (VAF) of 46%. Additional somatic variants with low VAFs were identified in *PIK3CA*, *APC*, and *TP53*. No significant CNVs were observed.

**FIGURE 2:**
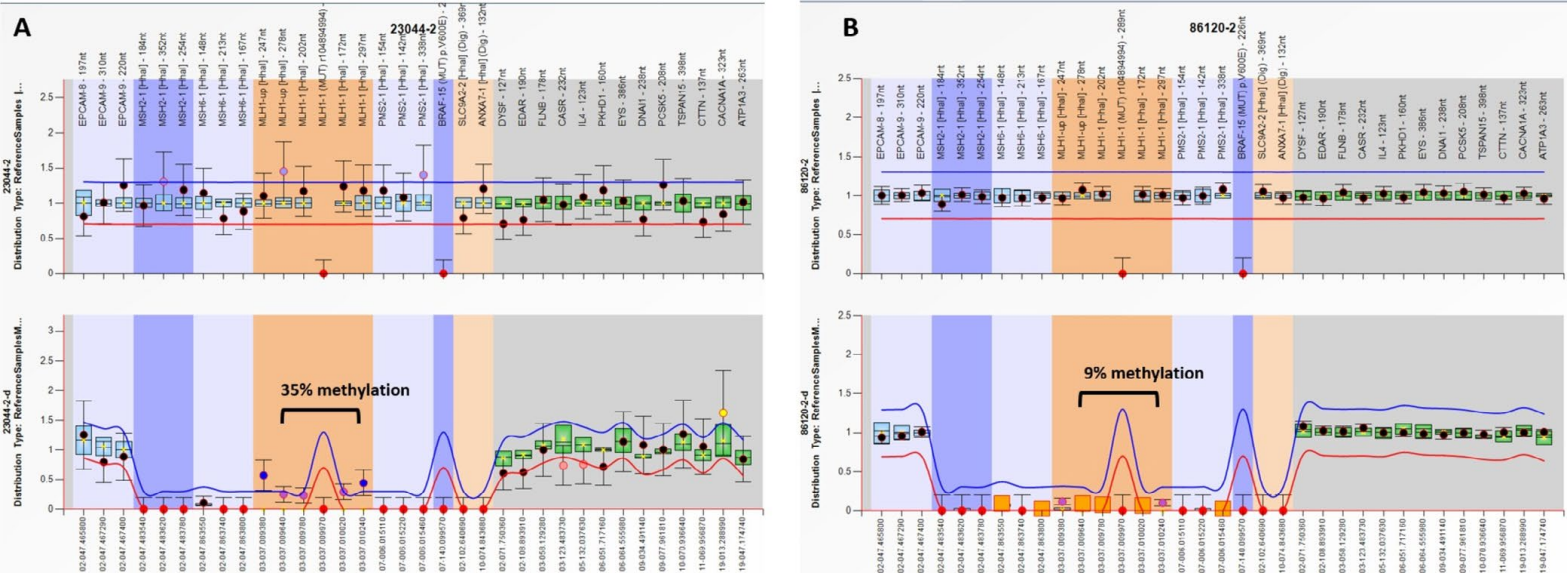
Increased methylation levels in all five MLH1 promoter probes tested in tumor tissue as shown by MS‐MLPA (A) and peripheral blood (B).

MS‐MLPA analysis using the coffalayser software of tumor DNA revealed increased methylation across all five *MLH1* promoter probes, with an average methylation level of approximately 35%, compared to 0% in control DNA (Figure 2A).

### 3.3 Germline Molecular Findings

Germline testing identified a heterozygous nonsense PV in *MSH6* (NM_000179.2:c.755C>G; p.Ser252Ter), classified as pathogenic according to the ACMG guidelines, ClinGen InSiGHT Hereditary Colorectal Cancer/Polyposis Expert Panel specifications, and ClinVar Expert Panel (Variation ID: 89566). No other pathogenic or likely PVs were detected in the remaining 12 genes analyzed, including *MLH1* and *PMS2*.

MS‐MLPA performed on two independent peripheral blood DNA samples from the patient demonstrated increased methylation of the *MLH1* promoter across all five probes, with an average methylation level of approximately 9%; compared to 0% in control DNA. This methylation increase was deemed statistically significant by Cofalyzer analysis (Figure 2B).

## 4 Discussion

This patient presented with a unique and diagnostically and clinically challenging scenario within the spectrum of LS. While the tumor exhibited loss of MLH1 and PMS2 expression by IHC, germline analysis failed to identify a PV in either *MLH1* or *PMS2*. Instead, a heterozygous truncating variant in *MSH6* (c.755C>G; p.Ser252Ter) was identified and confirmed to be inherited from the patient's father.

**FIGURE 3:**
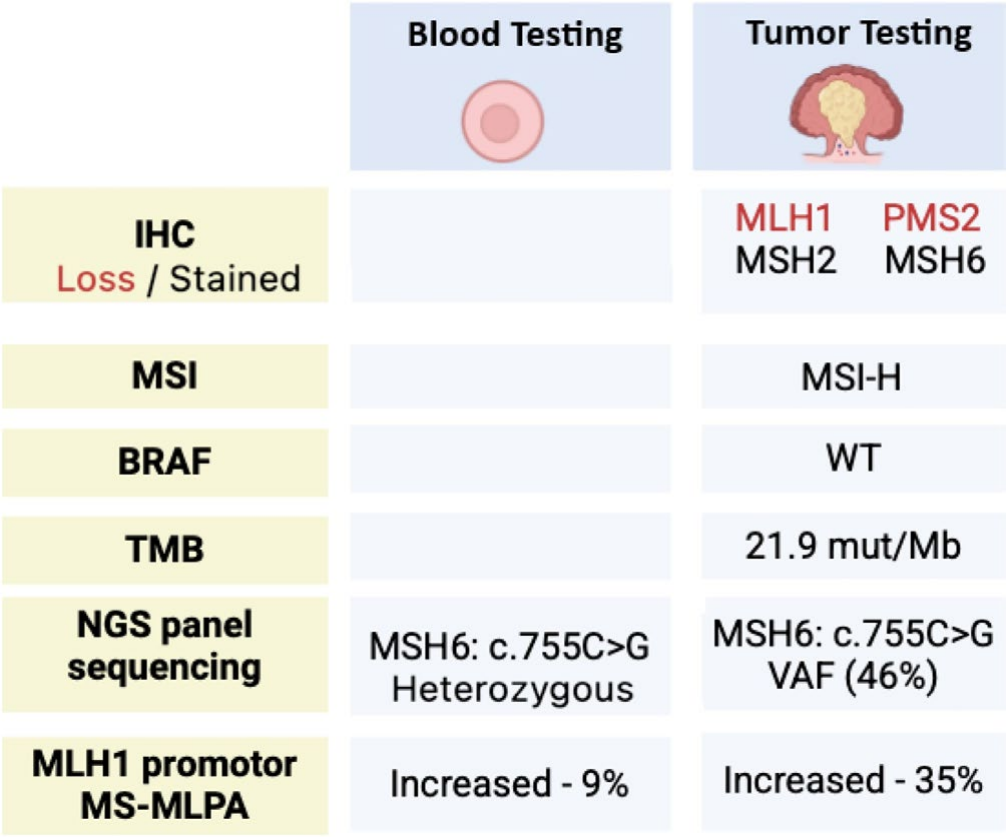
Comparative summary of immunohistochemical and molecular findings from tumor and germline testing. This figure illustrates the integrated diagnostic workup for germline and somatic colorectal cancer analysis.

Tumor testing in our patient revealed wild‐type *BRAF*. Paired tumor and germline testing failed to identify somatic mutations in *MLH1* or *PMS2*, but revealed a pathogenic *MSH6* variant in the tumor (VAF 46%). Given the discordance between the IHC profile and molecular findings—and considering the patient's young age, additional testing was pursued. MS‐MLPA confirmed statistically significant *MLH1* promoter hypermethylation in both blood and tumor DNA, supporting a diagnosis of mosaic constitutional *MLH1* epimutation (Figure 3).

The identification of both constitutional *MLH1* promoter hypermethylation and a germline pathogenic MSH6 variant in this patient raises the possibility of dual molecular contributions to the MMR‐deficient phenotype. While the IHC pattern supports loss of MLH1 expression, the tumor also harbors a pathogenic MSH6 variant at a variant allele frequency of 46%. The absence of a second somatic hit in MSH6 or detectable LOH limits definitive interpretation, but other mechanisms of inactivation—such as promoter methylation or copy‐neutral LOH—were not assessed and cannot be excluded. Therefore, we cannot establish a clear causal hierarchy between the two findings. It is plausible that both alterations contributed, either independently or in a synergistic manner, to the tumor's MMR deficiency.

The tumorigenic mechanism in individuals with constitutional *MLH1* epimutation is fundamentally distinct from that seen in cases of somatic *MLH1* promoter methylation. Somatic methylation typically leads to biallelic hypermethylation of the *MLH1* promoter in CRC, resulting in complete gene silencing, a process that is restricted to malignant cells and usually associated with older age at cancer diagnosis [2, 11]. In contrast, constitutional *MLH1* epimutation involves monoallelic *MLH1* promoter hypermethylation present in normal tissues, which inactivates one allele. A subsequent somatic event affecting the second allele leads to biallelic inactivation and loss of MMR function [2]. This tumorigenic process mimics the classic two‐hit mechanism of LS. Constitutional *MLH1* promoter hypermethylation, although rare, has been associated with high risk of early‐onset Lynch‐spectrum tumors, often in the absence of family history [11].

Our patient harbors two defective MMR alleles—one due to a germline *MSH6* mutation and the second via constitutional *MLH1* methylation—at least in mosaic distribution. Her clinical presentation at age 27, including CALMs, a resected scalp neurofibroma, and AVM, may partially overlap with some features seen in constitutional mismatch repair deficiency syndrome (CMMRD), the biallelic form of LS; these findings are clearly insufficient to make a diagnosis of CMMRD, but this unique combination raises the possibility of a digenic interaction between genetic and epigenetic alterations in MMR genes, potentially leading to a more severe phenotype. In light of this, we suggested that surveillance for our patient will follow a more precautious protocol to address the potential risk for extracolonic malignancies [12].

This report is limited by the absence of molecular characterization of the patient's neurofibroma, which could have provided further insights into the genetic or epigenetic basis of her phenotype. Additionally, the mosaic nature of the *MLH1* promoter hypermethylation constrains our ability to assess its distribution across tissues. Functional validation of a potential interaction between the *MLH1* hypermethylation and the *MSH6* variant was beyond the scope of this work.

## 5 Conclusion

This case underscores the critical importance of comprehensive molecular profiling in LS diagnostics, particularly in patients with discordant IHC and germline results and atypical clinical presentations. It also highlights the possible contribution of digenic mechanisms encompassing both germline and epigenetic alterations in modulating cancer risk and phenotype. Further research is warranted to explore the interplay between constitutional *MLH1* hypermethylation and MMR gene variants. This case exemplifies the complexity of hereditary cancer syndromes and the value of integrative multiomic diagnostics, including epigenetics, in uncovering novel molecular mechanisms.

## Author Contributions

All authors made a significant contribution to the work, whether that is in the conception, study design, execution, acquisition of data, analysis and interpretation.

## Ethics Statement

The study was conducted in accordance with the Declaration of Helsinki and approved by the Institutional Review Board of Rabin Medical Center (code 0847–22).

## Consent

The authors obtained written informed consent for genetic testing and anonymous publication of results from the patients.

## Conflicts of Interest

The authors declare no conflicts of interest.

## Data Availability

The data that support the findings of this study are available on request from the corresponding author. The data are not publicly available due to privacy or ethical restrictions.
